# Coaxial Bioprinting of Enzymatically Crosslinkable Hyaluronic Acid-Tyramine Bioinks for Tissue Regeneration

**DOI:** 10.3390/polym16172470

**Published:** 2024-08-30

**Authors:** Alma Tamunonengiofori Banigo, Laura Nauta, Bram Zoetebier, Marcel Karperien

**Affiliations:** Department of Developmental BioEngineering, Faculty of Science and Technology, TechMed Centre, University of Twente, Drienerlolaan 5, 7522 NB Enschede, The Netherlands; a.tamunonengioforibanigo@utwente.nl (A.T.B.); l.nauta@utwente.nl (L.N.); b.zoetebier@utwente.nl (B.Z.)

**Keywords:** bioink, hyaluronic acid-tyramine conjugates, coaxial bioprinting, enzymatic crosslinking, tissue regeneration

## Abstract

Three-dimensional (3D) bioprinting has emerged as an important technique for fabricating tissue constructs with precise structural and compositional control. However, developing suitable bioinks with biocompatible crosslinking mechanisms remains a significant challenge. This study investigates extrusion-based bioprinting (EBB) using uniaxial or coaxial nozzles with enzymatic crosslinking (EC) to produce 3D tissue constructs in vitro. Initially, low-molecular-weight dextran-tyramine and hyaluronic acid-tyramine (LMW Dex-TA/HA-TA) bioink prepolymers were evaluated. Enzymatically pre-crosslinking these prepolymers, achieved by the addition of horseradish peroxidase and hydrogen peroxide, produced viscous polymer solutions. However, this approach resulted in inconsistent bioprinting outcomes (uniaxial) due to inhomogeneous crosslinking, leading to irreproducible properties and suboptimal shear recovery behavior of the hydrogel inks. To address these challenges, we explored a one-step coaxial bioprinting system consisting of enzymatically crosslinkable high-molecular-weight hyaluronic acid-tyramine conjugates (HMW HA-TA) mixed with horseradish peroxidase (HRP) in the inner core and a mixture of Pluronic F127 and hydrogen peroxide in the outer shell. This configuration resulted in nearly instantaneous gelation by diffusion of the hydrogen peroxide into the core. Stable hydrogel fibers with desirable properties, including appropriate swelling ratios and controlled degradation rates, were obtained. The optimized bioink and printing parameters included 1.3% *w*/*v* HMW HA-TA and 5.5 U/mL HRP (bioink, inner core), and 27.5% *w*/*v* Pluronic F127 and 0.1% H_2_O_2_ (sacrificial ink, outer shell). Additionally, optimal pressures for the inner core and outer shell were 45 and 80 kPa, combined with a printing speed of 300 mm/min and a bed temperature of 30 °C. The extruded HMW HA-TA core filaments, containing bovine primary chondrocytes (BPCs) or 3T3 fibroblasts (3T3 Fs), exhibited good cell viabilities and were successfully cultured for up to seven days. This study serves as a proof-of-concept for the one-step generation of core filaments using a rapidly gelling bioink with an enzymatic crosslinking mechanism, and a coaxial bioprinter nozzle system. The results demonstrate significant potential for developing designed, printed, and organized 3D tissue fiber constructs.

## 1. Introduction

Three-dimensional bioprinting has demonstrated its advantages in the production of tissue-engineered structures with unique characteristics, which cannot be attained with traditional fabrication techniques [1]. Several 3D bioprinting techniques, including laser-based, stereolithography-based, inkjet-based, and extrusion-based (EB), with their own advantages and constraints, have been used to produce 3D tissue structures. EB bioprinting, the most common technique, is considered based on its broad range of available biomaterials with appropriate viscosities, a variety of crosslinking mechanisms, and compatibility with cells that can be used as so-called bioinks [2,3,4]. Uniaxial [5,6,7] and coaxial [8] EB bioprinting involve the extrusion of single or multiple biomaterials, cells, and bioactive molecules to print 3D constructs. Coaxial bioprinting is a cutting-edge field in TE designed for controlled concentric extrusion of multiple materials, or printing of complex structures in a shell/core configuration. Despite the advancement in EB bioprinting techniques, it remains a challenge to develop suitable bioinks. 

Hyaluronic acid (HA) is a promising polymer for making bioinks due to its viscoelastic and shear-thinning properties [9], excellent biocompatibility [10], and biodegradability [11]. HA can be easily modified chemically to enhance its properties or to introduce specific functionalities required for bioprinting and tissue engineering, e.g., by attaching functional groups for crosslinking [12]. HA is a natural component of cartilage and other connective tissues. As a paramount naturally derived polymer, HA has been utilized in the clinics for more than 35 years, which makes it a useful tool in the medical field [13]. Past research has demonstrated HA’s effectiveness in numerous medical and bioprinting applications. HA has been utilized in cartilage repair [14,15], wound healing [16], ophthalmology [16], drug distribution, and tissue engineering [17]. Specifically, HA’s use in 3D bioprinting [18,19,20,21,22,23,24,25] has shown significant promise due to its biocompatibility, tunable properties, and ability to support cell viability and function within printed constructs.

In 3D bioprinting, pristine HA solutions flow after deposition; in other words, shape retention and fidelity would require crosslinking mechanisms, such as UV crosslinking or enzymatic crosslinking mechanisms [26]. A typical example is the use of methacrylated hyaluronic acid (HA-MA), which crosslinks using UV light to produce scaffolds in both uniaxial [26] and coaxial [23,24,25] bioprinting of cartilage tissue. Despite the positive outcomes, the presence of the photo-initiator and the UV crosslinking mechanism can negatively affect cell viability and function. 

To overcome the limitations of UV light, enzymatic crosslinking of polymer-phenol conjugates in the presence of horseradish peroxidase (HRP) and hydrogen peroxide (H_2_O_2_) [7] can be explored to produce injectable hydrogels [27,28] and 3D bioprinted constructs [5,6]. HRP catalyzes the crosslinking reaction in the presence of H_2_O_2_, producing hydrogels with tunable gelation rates and crosslinking densities [29]. While HRP does not adversely affect cell fate, H_2_O_2_ can be a significant concern for cytocompatibility [30]. As long as the H_2_O_2_ concentration is kept low while having relatively fast crosslinking kinetics (tunable by HRP concentration), the H_2_O_2_ is quickly consumed for crosslinking of the phenolic moieties, keeping the exposure of the cells to a minimum and avoiding cytotoxic effects. Previous studies have shown the effectiveness of this crosslinking system with good cell viability, showing the cytocompatibility of the hydrogels [14,31]. 

Low-molecular-weight dextran-tyramine and hyaluronic acid-tyramine conjugates (LMW Dex-TA/HA-TA), in a ratio of 50:50, have been used in some studies stimulating cell attraction and chondrogenesis above other positive outcomes [14,15]. This hydrogel platform (LMW Dex-TA/HA-TA) is excellent for injectability but has not been tested for bioprintability. Therefore, the aim of this study was to explore this biomaterial platform as a bioink. Our initial strategy was drawn from the work of Petta et al. [5,6,7,13], where they considered pre-crosslinking to increase viscosity while retaining good shear-thinning properties and reducing the gelation time to print 3D constructs.

Here, we describe the optimization and production of enzymatic pre-crosslinked LMW Dex-TA/HA-TA bioink. The rheological properties of the bioink were tunable by varying the polymer concentrations and H_2_O_2_/TA ratios. A viscous ink could be obtained and extruded through a uniaxial nozzle, after which the printed structure could be further crosslinked by incubation in a H_2_O_2_ solution. Since pre-crosslinking proved highly variable, yielding inconsistent prints, we instead explored increasing the molecular weight of hyaluronic acid-tyramine conjugates to increase the viscosity of the bioink. In addition, we reduced the post-printing crosslinking time by inducing crosslinking at the same time as bioink extrusion through a coaxial printing configuration. The viscoelastic properties of HMW HA-TA were tunable by varying the polymer concentrations. This bioink was used to print filaments using a coaxial extrusion-based bioprinting process, in which the polymer conjugate mixed with horseradish peroxidase was present in the inner nozzle and the outer nozzle contained a sacrificial bioink mixed with hydrogen peroxide, serving as a support structure. Diffusion of hydrogen peroxide into the inner core resulted in fast outside in gelation of the polymer conjugates. Using this method, we were successfully able to print cell-laden filaments with good cell survival up to seven days. 

## 2. Materials and Methods

### 2.1. Materials 

Low- and high-molecular-weight (LMW and HMW) hyaluronic acid (HA) sodium salts from *Streptococcus equi*, with an average molecular weight of 27 kDa and 2.0–2.2 MDa (pharmaceutical grade), were purchased from Contipro, Dolní Dobrouč, Czech Republic. Dextran (Dex) 40 EP (40 kDa, pharmaceutical grade) was purchased from Pharmacosmos, Denmark. The 4-(4,6-dimethoxy-1,3,5-triazin-2-yl)-4-methylmorpholinium chloride (DMTMM, 97%) was obtained from Fluorochem Ltd., Hadfield, UK. Dulbecco’s Modified Eagle’s Medium (DMEM) was purchased from Thermo Fisher Scientific, The Netherlands. Tyramine (99%), DMF (anhydrous, 99.8%), LiCl (99.0%), p-nitrophenyl chloroformate (PNC, 96%), pyridine (anhydrous, 99.8%), DMSO-d_6_ (99.9%), NaCl (≥99.0%), tyramine hydrochloride (TA∙HCL, 99%), D_2_O (99.9 atom %D), horseradish peroxidase (HRP, 250 U/mg), and hydrogen peroxide (H_2_O_2,_ 30%) were purchased from Sigma-Aldrich, Schnelldorf, Germany. Ethanol (≥99.9%) and diethyl ether (≥99.7%) were purchased from Merck, Kenilworth, NJ, USA. Milli-Q water was used, from Milli-Q Advantage A10 system (Merck KGaA, Darmstadt, Germany), equipped with a 0.22 μm Millipak^®^-40 Express filter. Chemicals were used without further purification.

### 2.2. Methods 

#### 2.2.1. Synthesis of Hyaluronic Acid-Tyramine (HMW and LMW HA-TA) and Dextran-Tyramine (Dex-TA)

Hyaluronic acid-tyramine (LMW and HMW) was synthesized by one-step amidation of the HA carboxyl groups with tyramine, as previously described by Petta et al. [7] and D’Este et al. [27]. The detailed polymer syntheses of LMW and HMW HA-TA and Dex-TA are described in the Supplementary Materials: Protocol No. 1. We prepared HMW HA-TA with two substitution degrees, i.e., 5.5 and 11. The DS of LMW HA-TA was 10. The degree of substitution (DS) of hyaluronic acid is given as the percentage of COOH groups modified in hyaluronic acid (i.e., per 100 disaccharide units).

Dextran-tyramine was synthesized by the activation of dextran with PNC and subsequent aminolysis with tyramine, adapted from Ramirez et al. [28], and can be found in Supplementary Materials Protocol 1.

#### 2.2.2. Hydrogel Bioink Solution Preparation

Preparation of Enzymatically Pre-Crosslinked LMW Dex-TA/HA-TA Bioink:

LMW Dex-TA (DS 13) and HA-TA (DS 10) were dissolved in PBS. Afterward, the solutions were mixed and incubated with HRP overnight at 4 °C. Pre-crosslinking was initiated by adding varying molar ratios of H_2_O_2_/TA, ranging from 0.025 to 0.047. The final concentrations were 5 or 10% *w*/*v* polymer, and 1–3 U/mL HRP. 

Preparation of HMW HA-TA Bioink:

HMW HA-TA was dissolved in PBS to achieve final concentrations of 1.3, 1.8, or 2.2% *w*/*v*. These solutions were incubated with 5.5 U/mL HRP overnight at 4 °C to ensure thorough mixing.

#### 2.2.3. Crosslinked Hydrogel Formation

Cylindrical (8 mm diameter and 1.5 mm height) hydrogel samples were produced with the use of a PTFE mold, as described by Fu et al. [14] and shown in Supplementary Materials Figure S1. HMW HA-TA was dissolved in PBS (1.3, 1.8, or 2.2% *w*/*v*) and incubated in the presence of HRP, at a final concentration of 5.5 U/mL, overnight at 4 °C. Then, it was crosslinked by the addition of freshly prepared H_2_O_2_ (0.0028–0.0092%; Supplementary Materials Table S1) at a 2:1 TA:H_2_O_2_ molar ratio and transferred into the PTFE mold using a 1 mL positive displacement pipette immediately after mild magnetic stirring. 

#### 2.2.4. Rheological Characterization

The rheological properties of the bioinks and corresponding hydrogels were measured using an MCR 301 rheometer (Anton–Paar) equipped with Peltier temperature control (C-PTD200). For viscosity measurements, parallel plates (ø 25 mm) with gap heights of 1 mm for HMW HA-TA, and double-gap configuration for LMW Dex-TA/HA-TA, were used. The viscosity of the non-crosslinked LMW Dex-TA/HA-TA bioinks was measured at 20 °C (±0.1 °C), while the HMW HA-TA bioinks were measured at 25 °C (±0.2 °C).

The viscoelastic properties of the corresponding hydrogels were examined using parallel plates of ø 8 mm. Measurements were conducted at 20 °C (±0.2 °C) or 25 °C (±0.2 °C) under an initial normal force of 0.05 N. The tests were performed within the linear viscoelastic (LVE) range, at a strain of 0.5% and a frequency of 1.0 Hz. Prior to measurement, the hydrogel samples were equilibrated in 1 mL PBS at 4 °C for 24 h.

For each formulation of HMW HA-TA (1.3%, 1.8%, and 2.2% *w*/*v*), a minimum of three bioink or hydrogel samples were measured to ensure reproducibility and accuracy of the data.

#### 2.2.5. Uniaxial Extrusion Bioprinting of LMW Dex-TA/HA-TA 3D Structures

LMW Dex-TA/HA-TA filaments were printed without cells using a conical 22 G nozzle attached to an Inkredible+ 3D Bioprinting System (Cellink). The bioprinting test procedure and overall G-Code generation process are available upon request. Filaments of 10 mm were printed with different printing pressures (10, 50, 75, 150, and 300 kPa) and speeds (2.5, 10, and 25 mm/s) in a polystyrene petri dish for printing assessment. The printing assessment focused on filament spread and diameter variation. 

Next, 3D printing potential was demonstrated by producing a hollow cylinder of 5 mm in diameter with the use of a 26 G straight nozzle, operated at a pressure of 250 kPa with a bioink consisting of LMW (5% *w*/*v*) Dex-TA/HA-TA at 0.040 H_2_O_2_/TA, mixed with 1 U/mL HRP. 

#### 2.2.6. Swelling Ratio Measurement of HMW HA-TA Hydrogel Samples

The hydrogel samples were incubated in 1 mL PBS at 37 °C after preparation, as described above (Hydrogel Formation Section). 

The weights of the swollen hydrogel samples (W_w_) were measured after 24 h, and the samples were lyophilized afterward for 48 h. Then, the lyophilized hydrogel samples were weighed to obtain the dry weight (W_d_). Three hydrogel samples were measured for each polymer formulation (concentration) of HMW HA-TA.

The swelling ratio was defined as the ratio of the water content ($w_{w}-w_{d}$) divided by the polymer content (dry weight, $w_{d}$), as in Equation (1) [14,31]:

(1) $$Swelling\;ratio=\frac{w_{w}-w_{d}}{w_{d}}$$

#### 2.2.7. Enzymatic Degradation of HMW HA-TA Hydrogel Samples

The prepared hydrogel samples were incubated in 1 mL PBS at 4 °C for 24 h and weighed to determine their initial masses (w_i_). For the hydrogel degradation, we incubated the HMW HA-TA hydrogel samples in 1 mL of either 2.5 U/mL or 5 U/mL hyaluronidase in PBS, which was refreshed daily. The change in weight of the HMW hyaluronic acid-based hydrogel samples was measured during incubation at 37 °C for 15 days (w_c_). The percentage of the remaining hydrogel samples was determined with the use of Equation (2) [31]:

(2) $$Degradation\;ratio\left(\%\right)=\frac{w_{c}}{w_{i}}\times 100\%$$

#### 2.2.8. Cell Culture and Expansion

Bovine primary chondrocytes (BPCs) were expanded in a chondrocyte proliferation medium (Dulbecco’s Modified Eagle’s Medium (DMEM); Gibco, Billings, MT, USA), supplemented by 10% fetal bovine serum (FBS; Sigma S0615, Lot No. 0001652821), 0.2 mM ascorbic acid 2-phosphate (Sigma), 0.4 mM proline (Sigma), 1× non-essential amino acids (Gibco), 100 U/mL penicillin, and 100 μg/mL streptomycin (Invitrogen, Carlsbad, CA, USA). The medium was refreshed twice a week, and cells were used for experiments at 80% confluency and in passage 3. 

The 3T3 fibroblasts were expanded in a fibroblast proliferation medium (Dulbecco’s Modified Eagle’s Medium (DMEM); Gibco, Billings, MT, USA), supplemented by 10% fetal bovine serum (FBS; Sigma S0615, Lot No. 0001652821), 100 U/mL penicillin, and 100 μg/mL streptomycin (Invitrogen, Carlsbad, CA, USA). Then, 2-mercaptoethanol (1.4 µL of a 50 mM solution per mL of culture medium) was freshly added to the culture medium immediately before pipetting into the culture flask. The medium was refreshed twice a week, and cells were used for experiments at 80% confluency in passage 5.

#### 2.2.9. Coaxial Bioprinting Test and Printing of (Cell-Laden) Core Filaments

Shell-core filaments were printed with and without cells (BPCs or 3T3Fs) using a custom-made coaxial nozzle. The coaxial bioprinting test procedure and overall G-Code generation process are described in the Supplementary Materials: Protocol No. 2 and 3. The coaxial nozzle had an inner core (IC) nozzle of 22 G (0.4 mm (ID) and 0.7 mm (OD)), and an outer shell (OS) nozzle of 14 G (1.6 mm (ID) and 2.2 mm (OD)), respectively. This coaxial nozzle was attached to an Inkredible + 3D Bioprinting System (Cellink) comprising two printheads with independent pneumatic controllers (<150 kPa) and temperature controllers (<40 °C; Figure S2). The (bio)ink composites were loaded in a 3 mL print cartridge before bioprinting. For the outer shell, 27.5% *w*/*v* Pluronic F127 dissolved in H_2_O, containing 0.1% H_2_O_2_, was used as the support material.

For non-cell-laden core filament printing, the formulations of HMW HA-TA and HRP were used in the inner core (IC) for coaxial printing. 

For cell-laden core filament printing, the cells (1 × 10^6^ cells/mL) were suspended in the HMW HA-TA prepolymer solutions of different concentrations with 5.5 U/mL HRP and loaded into the inner core (IC) print cartridge. 

During the printing process, both the IC and OS solutions were extruded through the coaxial nozzle connected to the Inkredible + 3D Bioprinter. The coaxial (bio)printing was carried out with an extrusion pressure ranging from 35 to 110 kPa on the inner core material and a fixed extrusion pressure of 80 kPa on the outer shell material. The printing speed was fixed at 300 mm/min and the bed temperature was 30 °C.

#### 2.2.10. Pluronic F127 Shell Removal

After printing in a 60 mm diameter × 15 mm height glass petri dish, the shell-core filaments were left on the warm (30 °C) printing bed for 1 min to completely crosslink. Then, 1 mL PBS (at room temperature) was transferred to the petri dish containing the shell-core filaments and incubated on a platform at room temperature for 1 min. F127 was completely removed from the shell material, leaving the core filaments only. The core filaments were analyzed macroscopically and microscopically. 

#### 2.2.11. Compression Test

Compression testing was performed on the cylindrical HMW HA-TA hydrogel samples (described in the Hydrogel Formation Section) with the use of the HR 20 Discovery Hybrid Rheometer-TA Instruments (USA). The hydrogels underwent a single compression cycle with a maximum strain of 30% using a compression speed of 0.05 mm/s. The compression tests were conducted at room temperature, and at least three specimens were tested for each formulation

#### 2.2.12. Live/Dead Staining

The effect of the bioink’s composition on cell viability was studied using live/dead assay. On days 0, 1, 3, and 7, the cultured control samples and printed core filaments were stained with Alexa Fluor-488 Calcein and Alexa Fluor-568 Ethidium homodimer using the live/dead assay kit (Invitrogen), according to the manufacturer’s instructions. The concentration of Alexa Fluor-488 Calcein for printed core filaments was increased 4 times for better visualization. 

Fluorescent confocal microscopy (Laser Scanning Microscope 880, Zeiss, Oberkochen, Germany) traced fluorescently labeled BPCs and 3T3 Fs in the different bioprinted cell-laden core filaments and was used to assess the cultured cell-laden core filaments. The objective EC Plan-Neofluar 10x/NA 0.3 was used for this study. Visualization from a single point or z-stacking images was performed to confirm the homogeneous distribution of the cells in the bioink. The single and Z-stack images were randomly selected from different areas of each sample. As a result, living cells showed fluoresce green and the nuclei of dead cells were red. FIJI 2.14.0 software was used for cell counting. The cell viability was calculated by the percentage of live cells (green) in the total cells (green + red) from each area. Values represent the mean ± standard deviation of at least 3 biological replicates. 

#### 2.2.13. Phalloidin and DAPI Staining 

The assessment of the cell shape on BPCs after coaxial bioprinting was carried out. On day 7, the cultured cell-laden core filaments were cut into slices with a surgical scalpel, fixed with 4% *w*/*v* paraformaldehyde, permeabilized with 0.5% *v*/*v* Triton X 100, and stained with 0.25% Alexa Fluor-488 Phalloidin (F-actin) as well as 1% Alexa Fluor-405 DAPI. 

The same Confocal Laser Scanning Microscope with objective Plan-Apochromat 20x/NA 0.80 was used for this study. The single images were randomly selected from different areas of each sample, and these images were processed using FIJI software. The cell shape was captured by the presence of actin filament (green) and the nucleus (blue) from each area. These images were processed using FIJI software.

## 3. Results and Discussion

### 3.1. LMW Dex-TA/HA-TA Bioink Properties and Bioprinting Process

Although LMW Dex-TA/HA-TA hydrogel precursors have a good injectability, the viscosity is too low, and the gelation time is too long to successfully print 3D constructs. To increase the viscosity, we introduced partial pre-crosslinking of the tyramine conjugated polymers with a small quantity of H_2_O_2_ in the presence of a fixed amount of HRP. Here, 10% *w*/*v* Dex-TA/HA-TA, prepared from Dex-TA batches with different degrees of substitution of tyramine, was mixed with 3 U/mL HRP and 0.0041% H_2_O_2_, resulting in ratios of H_2_O_2_/TA of 0.022–0.028 to produce viscous polymer solutions (Supplementary Materials Figure S3). Some Dex-TA batches yielded a strong shear-thinning behavior and viscosity comparable to the Cellink Start bioink, which served as the control. The solution with the lowest viscosity and poor shear-thinning properties, based on Dex-TA batch N/DS13, was further optimized. This Dex-TA batch, with the same HRP and polymer concentrations, was mixed with H_2_O_2_ in H_2_O_2_/TA ratios ranging from 0.025 to 0.033 to yield a viscosity profile and shear-thinning behavior similar to Cellink Start (Supplementary Materials Figure S4a). Then, from the same Dex-TA batch, a lower polymer concentration (5% *w*/*v*) with H_2_O_2_/TA ratios ranging from 0.033 to 0.042 was also tuned to a viscosity similar to Cellink (Supplementary Materials Figure S4b). Further experiments were conducted with the use of 5% *w*/*v* LMW Dex-TA (batch N/DS13)/HA-TA hydrogel with 1 U/mL HRP and various H_2_O_2_/TA ratios. We studied the effect of H_2_O_2_ on the shear viscosity. 

**Figure 1:**
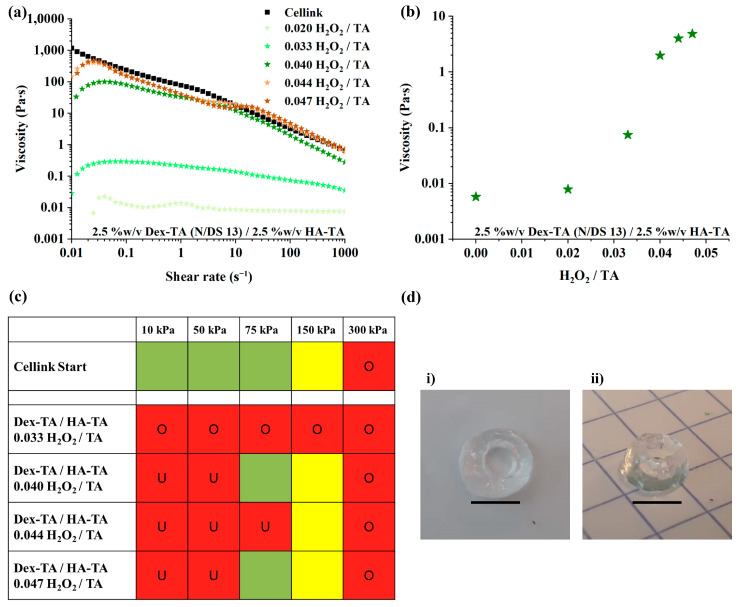
Rheological analysis and printing conditions of 5% *w*/*v* LMW Dex-TA/HA-TA (Dex-TA: N/DS 13) with 1 U/mL HRP. (**a**) Viscosity during a shear rate sweep from 0.01 s^−1^ to 1000 s^−1^ of LMW Dex-TA/HA-TA solutions pre-crosslinked at different H_2_O_2_/TA ratios, represented by the numbers in the legend. (**b**) Viscosity at 100 s^−1^ shear as a function of the H_2_O_2_/TA ratio. As shown in the plot, viscosity was positively correlated with an increase in the H_2_O_2_/TA ratio. At higher H_2_O_2_/TA ratios of >0.04, the viscosity increase seemed to reach a plateau. (**c**) Printing studies of bioinks at different printing pressures and a single nozzle of 25 G. The printing quality is identified with distinct colors: good (green, <200% filament spread and <10% diameter variation), moderate (yellow, 200–300% filament spread and >10% diameter variation), or poor (red, divided into under-extrusion (U) and over-extrusion (O)) printability, depending on the criteria, as shown in the legend. (**d**) The 3D-printed hollow cylinder (**i**) top view and (**ii**) side view. The grid lines in image (**ii**) comprise of 5 mm × 5 mm (scale bars represent 5000 µm).

Results in Figure 1a,b showed that an increase in H_2_O_2_ led to an increase in viscosity, levelling off at higher H_2_O_2_ levels (H_2_O_2_/TA ratio of 0.040–0.047). Based on the rheological studies, LMW Dex-TA/HA-TA with 0.033–0.047 H_2_O_2_/TA were selected for printability studies, and the results are presented in Figure 1c and detailed in Table S2. LMW Dex-TA/HA-TA with an H_2_O_2_/TA ratio of 0.040–0.047 could be extruded at 75 kPa, but LMW Dex-TA/HA-TA with a ratio of 0.044 could not. LMW Dex-TA/HA-TA with an H_2_O_2_/TA ratio of 0.040 and 1.0 U/mL HRP, having a viscosity of 0.694 Pa∙s at 100 s^−1^, was used to 3D-print a hollow cylinder with a 26 G straight nozzle and a pressure of 250 kPa (shown in Figure 1d). 

Despite the fact that LMW Dex-TA/HA-TA 0.040 could print the hollow cylinder and had similar viscosity and shear-thinning properties as Cellink Start, the viscosity and printing of pre-crosslinked LMW Dex-TA/HA-TA were very inconsistent. Therefore, the use of pre-crosslinked LMW HA-TA/Dex-TA solutions was abandoned, and additional improvements were investigated.

### 3.2. HMW HA-TA Bioink Properties and Bioprinting Process

Petta et al. [5,6,7,13] used medium-molecular-weight hyaluronic acid-tyramine (MMW HA-TA: 280 kDa; DS 14.5%) with higher viscosity and good shear-thinning properties relative to LMW HA-TA to print 3D constructs. This clearly showed that there was a significant difference in the rheological properties of LMW, MMW, and HMW HA, which will be relevant for 3D bioprinting. Based on that, our second strategy involved the development of enzymatically crosslinkable HMW HA-TA with unique properties, including good shear-thinning properties, high viscosity, shear recovery, biodegradability, and chondroprotection [7,13,32,33,34]. 

**Figure 2:**
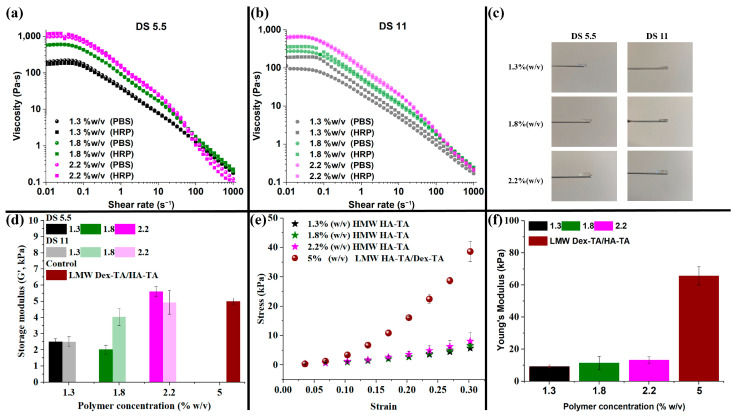
Shear-thinning and mechanical properties of HMW HA-TA bioinks and corresponding hydrogels. Shear-thinning profile of HMW HA-TA with (**a**) DS 5.5 and (**b**) DS 11 at polymer concentrations of 1.3, 1.8, and 2.2% *w*/*v*. (**c**) Hydrogels produced with 5.5 U/mL HRP and a 0.5 H_2_O_2_:TA molar ratio. (**d**) Storage modulus of all hydrogel compositions, including a 5% *w*/*v* LMW Dex-TA/HA-TA control hydrogel. (**e**) Stress–strain curves of DS 5.5 hydrogels compared with a 5% *w*/*v* LMW Dex-TA/HA-TA control hydrogel. (**f**) Young’s modulus of the cylindrical hydrogels.

Then, rather than printing in a crosslinking bath, we explored coaxial bioprinting to develop a one-step printing process [8,35]. HMW HA-TA was synthesized with a DS of 5.5 and 11 (Supplementary Materials Figure S5a,b). Our bioink in the inner core (IC) print cartridge, consisting of HMW HA-TA and horseradish peroxidase (HRP), showed shear thinning (Figure 2), which is an essential property for a bioink depositing through a nozzle [36]. Interestingly, an increase in the viscosity profile was observed for the lower concentrations of HMW HA-TA DS 11 upon the addition of HRP, while other bioink compositions retained their viscosity profile after the addition of HRP (Figure 2b). The formulations possessed high viscosities of 100 up to 1000 Pa·s at the Newtonian plateau (0.01–0.1 s^−1^), preventing bioink spread and filament collapse, while aiding in the shape fidelity of the printed constructs. Viscosities for both DS 5.5 and 11 formulations were below 45 Pa·s within the shear rate range of 10 to 30 s^−1^, which is essential for optimal extrusion and print quality [6]. More so, above a shear rate of 30 s^−1^, the viscosity of all formulations reduced to less than 10 Pa·s, which facilitated smooth and efficient extrusion through the nozzle. The viscosity profile was beneficial for both good-quality prints and the reduction in shear stress [25] on encapsulated cells during printing. By decreasing shear-induced damage [25], the printing conditions helped to ensure high cell viability within the core filaments, which is important for successful bioprinting applications. For each DS, an increase in polymer concentration from 1.3% *w*/*v* to 2.2% *w*/*v* led to an increase in viscosity at the Newtonian plateau, with DS 5.5 exceeding the values of DS 11. This could be explained by the hydrophobic nature of the grafted tyramine, yielding a more hydrophobic polymer by increasing the DS. H_2_O_2_ was used in the outer shell as a crosslinking agent. To control the inward diffusion of H_2_O_2_ into the bioink, the H_2_O_2_ was included in a sacrificial ink (Pluronic F127). Here, 27.5% *w*/*v* Pluronic F127 was chosen after a preliminary cell viability study (results not shown), and the shear-thinning properties were determined (Supplementary Materials Figure S6). The 27.5% *w*/*v* Pluronic F127 had a viscosity of more than 10 kPa∙s at 0.01 s^−1^, giving temporary structural support to the printed coaxial construct, maintaining the shape and integrity of the printed construct during bioprinting and avoiding collapse as the IC bioink was being extruded. More so, it was easily removed or dissolved by changing the temperature without endangering the IC filaments. The rheological measurements were performed on cell-free bioinks; however, the addition of cells could reduce the viscosity of the solution. 

**Figure 3:**
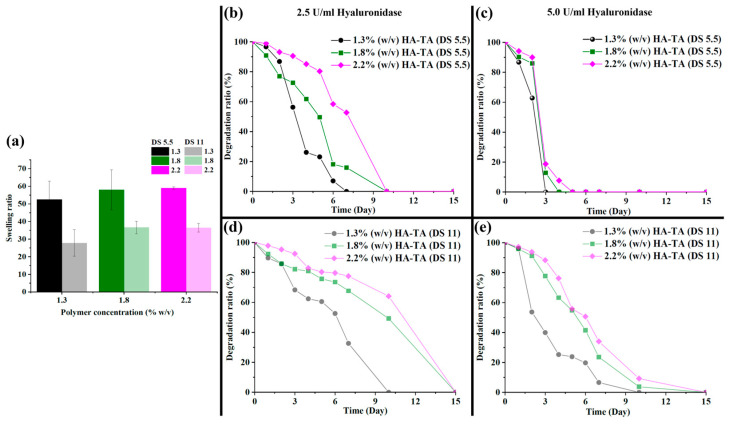
Physical properties of the HMW HA-TA hydrogels. (**a**) Swelling ratio of the HMW HA-TA hydrogels. (**b**) Degradation of the HMW HA-TA hydrogels with (**b**) DS 5.5 in 2.5 U/mL hyaluronidase, (**c**) DS 5.5 in 5 U/mL hyaluronidase, (**d**) DS 11 in 2.5 U/mL hyaluronidase, and (**e**) DS 11 in 5 U/mL hyaluronidase.

The gelation of the bioinks was tuned to be nearly instantaneous when exposed to H_2_O_2_. This was required for coaxial bioprinting of the core filaments. Due to the nearly instantaneous gelation, no gelation time could be determined. Figure 2d displays the storage moduli (G’) of all crosslinked bioink formulations. The cylindrical hydrogels that were produced for rheology are shown in Figure 2c. Overall, independently of DS, the storage modulus increased with an increase in the polymer concentration, with 1.8% *w*/*v* HMW HA-TA DS 5.5 being an exception. This could be a result of inhomogeneity, introduced by the nearly instantaneous crosslinking. The Young’s modulus obtained from the stress/strain curves in Figure 2e was found to increase with the increasing HMW HA-TA concentration (1.3 to 2.2% *w*/*v*). Even though the storage moduli of the crosslinked bioinks corresponded well with our 5% *w*/*v* LMW sample, as discussed in Section 3.1, the Young’s modulus of the 5% *w*/*v* LMW hydrogel (66.1 kPa) exceeded the crosslinked HMW bioinks by 5–10-fold (Figure 2f). This result clearly shows that 1.3 to 2.2% *w*/*v* HMW HA-TA crosslinked bioink samples were very well able to store energy elastically; however, they were not as stiff as 5% *w*/*v* LMW HA-TA/Dex-TA. The 5% *w*/*v* LMW HA-TA/Dex-TA served as a comparison to better understand our bioinks’ mechanical properties and interpretation of the data. Interestingly, the increase in crosslinking (DS 11 vs. DS 5.5) did not have a significant effect on the storage modulus. However, it almost halved the swelling ratio (Figure 3a), shrinking the formed gel discs after preparation. The swelling ratio increased with an increase in the HMW HA-TA concentration (1.3, 1.8, or 2.2% *w*/*v*). This may be because the hydrogels are made of polyelectrolytes, and they swell with an increase in polymer concentration based on the charge repulsion among polymer chains. In the degradation test, the degradation rate was clearly dependent on the polymer concentration, DS, and hyaluronidase concentration (Figure 3b–e). A higher polymer concentration as well as higher DS (corresponding to a higher crosslinking) resulted in a slower degradation [31]. A higher hyaluronidase concentration reduced the degradation time and steepened the degradation profile. These findings are expected to extend to printed filaments, as degradation in these hydrogels is unlikely to be diffusion limited. Furthermore, in terms of long-term stability, it is anticipated that the integrity of the printed constructs will be maintained, as the extracellular matrix (ECM) produced by the cells is expected to gradually replace the hydrogel matrix. Based on the favorable low shear viscosity and swelling ratios of inks with a DS of 5.5, these inks were used for the printing experiments. 

**Figure 4:**
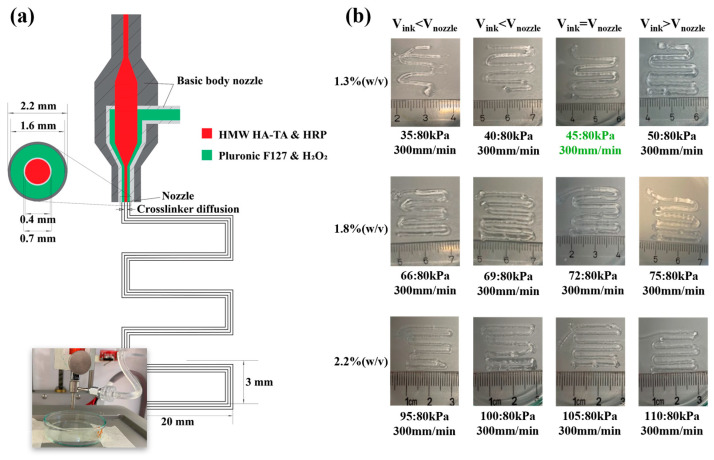
(**a**) Schematic representation of the coaxial 3D-printing system with a cross-sectional view of the coaxial nozzle tips and programmed pattern designed for printing. (**b**) Coaxially printed core filaments of 1.3, 1.8, and 2.2% *w*/*v* HMW HA-TA with different inner core pressures, a fixed outer shell pressure (80 kPa), and the same printing speed (300 mm/min); conditions for a speed match are indicated in green.

In Figure 4a, a schematic representation of the coaxial 3D-printing system is depicted, providing a cross-sectional view of our produced coaxial nozzle tips and programmed snake pattern designed for printing. The assessment of the printability of HMW HA-TA bioink at different concentrations using the designed coaxial nozzle is illustrated in Figure 4b, demonstrating successful printing with HMW HA-TA bioink (DS 5.5) containing 1.3, 1.8, and 2.2% *w*/*v*. The feasibility of one-step printing with a coaxial nozzle was investigated. The bioink contained in the syringe was connected to the inner core (IC), while Pluronic F127 was connected to the outer shell (OS) of the coaxial nozzle. Upon convergence at the tip of the nozzles, the HMW HA-TA ink underwent nearly instantaneous gelation, allowing for the printing of filaments in a snake pattern (Figure 4b) directly into a petri dish. Subsequently, the outer shell ink could be removed, leaving behind the printed structure.

For crosslinking during printing, the feeding rates of the H_2_O_2_-containing outer shell and the inner core ink should match within a suitable range (results not included) [36]. 

For printing optimization and replication, the HMW HA-TA core filament extrusion speed should match the printing speed (Figure 4b) [35,36]. Achieving this avoids under- or over-extrusion of inks (indicated by < or > symbols), consistency in material deposition, and accurate layer alignment. We obtained a speed match (=) with the 1.3% *w*/*v* HMW HA-TA core filament at 45:80 kPa (inner core:outer shell pressures) and a speed of 300 mm/min (highlighted in green, Figure 4b). 

**Figure 5:**
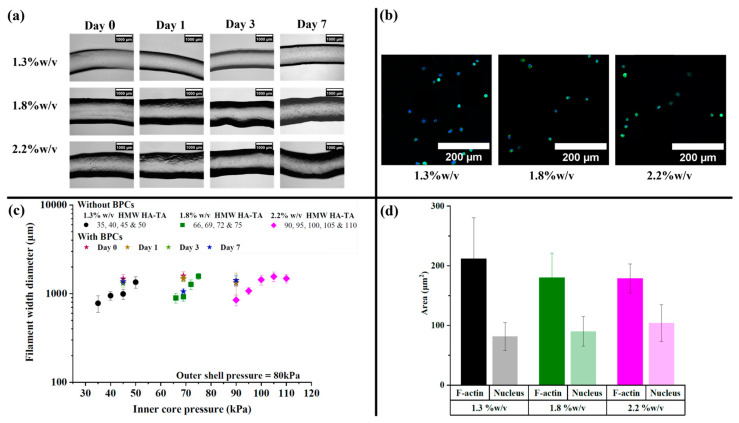
Core filaments with cells. (**a**) BPC-laden core filaments printed with 1.3, 1.8, and 2.2% *w*/*v* HMW HA-TA bioinks containing 5.5 U/mL HRP, printed at 45, 69, and 90 kPa, respectively (scale bars represent 1000 µm). (**b**) The BPCs were stained for F-actin and nuclei on day 7, revealing their rounded shape (scale bars represent 200 µm). (**c**) Quantified filament width of the core filaments with and without BPCs. (**d**) Quantified area of the rounded F-actin and nuclei.

The images of non-cell-laden and cell-laden core filaments produced using 1.3, 1.8, and 2.2% *w*/*v* HMW HA-TA bioinks are shown in Figure 4b and Figure 5a, respectively. The core filaments with bovine primary chondrocytes (BPCs) were produced with selected inner core pressures (Figure 5c), adjusted for ink viscosity, and assessed for cell viability on days 0, 1, 3, and 7. The cells remained round on day 7, and the area of the F-actin and nuclei was quantified (Figure 5b,d). Using HMW HA-TA ink, the outer diameter of the core filaments without cells increased with an increase in the inner core pressure for each polymer concentration (Figure 5c). In addition, the diameter of the cell-laden filaments was larger compared to the non-cell-laden filaments when printed under the same pressure. 

**Figure 6:**
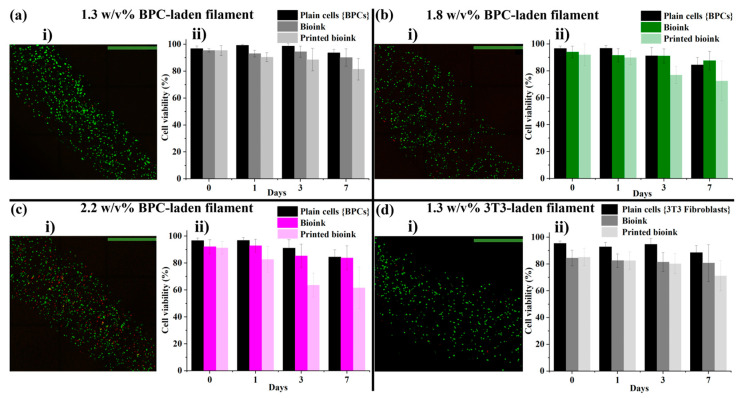
Core filaments with cells. (**ai**–**ci**) Confocal fluorescence images of printed BPC-laden filaments of 1.3, 1.8, and 2.2% *w*/*v* HMW HA-TA, respectively. (**di**) Confocal fluorescence images of printed 3T3 fibroblast-laden filaments of 1.3% *w*/*v* HA-TA (scale bars represents 800 µm). (**aii**–**dii**) Corresponding cell viability plots of plain cells (BPCs and 3T3 fibroblasts), extruded bioinks, and printed bioinks (crosslinked filaments).

The addition of cells caused changes in the overall bioink characteristics; particularly, the diluting effect of the volume fraction of the encapsulated cells and the residual volume of the culture medium in the centrifuged cell pellet [31] decreased the viscosity, especially for 1.8 and 2.2% *w*/*v* HMW HA-TA bioinks. Therefore, lower IC pressures (69 and 90 kPa, respectively) were used for coaxial bioprinting of the cell-laden core filaments. When using cell-laden bioink formulations, the cells were homogenously distributed inside the printed filaments (Figure 6(ai–di)). For chondrocyte-laden filaments, over 90% of the cells were viable in all conditions at day 0, and over 82% of the cells were still viable in the best conditions (1.3% *w*/*v* HMW HA-TA) at day 7 (Figure 6(aii–dii)). Further decreases in cell viability for 1.8 and 2.2% *w*/*v* HMW HA-TA core filaments were observed due to higher shear stresses during extrusion. Further investigation showed a reduction in cell survival when extruding the BPC-laden bioink through a nozzle. An additional reduction in cell viability was observed after the bioprinting process (Figure 6(aii–cii); corresponding confocal fluorescence images are presented in Supplementary Materials Figure S7).

Compared to earlier studies on BPC-laden hydrogels, the cell viability observed in the bioinks on day 7 (D7) was relatively high, indicating the potential for sustained long-term viability. For instance, Fu et al. reported a reduction in cell viability of 15% and 12% within the first 7 days for their 5 w/v% and 10 w/v% hydrogels, respectively. However, in the subsequent 14 days, the decline in cell viability stabilized, with further reductions limited to 5% and 0%, respectively [14].

Based on the BPC results, 3T3 fibroblast-containing HMW HA-TA core filaments were only printed with 1.3% *w*/*v*, yielding over 80% of the cells viable on day 0 and over 70% on day 7 (Figure 6(di,dii)). The drop in cell viability after extrusion shows that these cells were more affected by the shear stress (Figure 6(dii), with corresponding confocal fluorescence images in Supplementary Materials Figure S8 and corresponding bright-field images in Supplementary Materials Figure S9).

The core filament sizes after printing depend on parameters including the viscosity, nozzle diameter, bioink extrusion rate, printing pressure, crosslinking rate, and nozzle speed during printing [31,37]. The drop in cell viability after extrusion for 3T3 fibroblasts in the core filaments compared to chondrocytes on day 0 may be a result of the inherent sensitivity of fibroblasts to shear stress during the mixing of the bioink and bioprinting processes. Fibroblasts could be more susceptible to mechanical damage, whereas chondrocytes may show better resilience [38]. In addition, HMW HA-TA hydrogel is chondroprotective [39,40], contributing to the higher initial viability of chondrocytes [13]. Additionally, the effect of the molecular weight and concentration of HMW HA-TA could inhibit cell adhesion and proliferation [41,42,43], resulting in decreased viability.

## 4. Conclusions

In this study, we showed that although enzymatic pre-crosslinking of LMW Dex-TA/HA-TA resulted in appropriate viscosity and shear-thinning properties for bioprinting, this approach was not successful in obtaining reproducible bioinks and 3D-printed constructs. To overcome the shortcomings, we employed an HMW HA-TA-based bioink and a coaxial bioprinting system to print core filaments in one step. The use of Pluronic F127 as a sacrificial ink is a good approach to support and improve the print fidelity of the core filaments with good cell viability. The use of the enzymatic crosslinking mechanism in combination with coaxial printing enabled nearly instantaneous gelation, which was paramount for this study. Here, 1.3% *w*/*v* HMW HA-TA showed good shear-thinning behavior, viscosity, mechanical properties, degradation profile, swelling ratio, cellular performance, and bioprintability. 

In combination with the fast-gelling materials, a coaxial nozzle extruder is an invaluable means of producing core filaments as the first step to successfully printing fibrous tissues in the future. In vitro bioprinting of living fibers has several potential applications in tissue regeneration for biomedical purposes.

## Data Availability

The data presented in this study are available upon request from the corresponding author.

## Supplementary Materials

The following supporting information can be downloaded at: https://www.mdpi.com/article/10.3390/polym16172470/s1. Protocol 1: Synthesis protocols. Protocol 2: Coaxial printing of HMW HA-TA bioink. Protocol 3: G-Code for coaxial bioprinting. Table S1: Final concentration of HMW hyaluronic acid-based hydrogels and control. Table S2: Optimal rheological properties of pre-crosslinked LMW Dex-TA/HA-TA bioink, printing parameters, and results of 3D bioprinting assessment. Figure S1: Sketch of the Teflon mold used for hydrogel formation. Figure S2a: Inkredible+ 3D Bioprinting System (Cellink). Figure S2b: Zoom-in of the coaxial nozzle. Figure S3: The viscosity profile of 10% *w*/*v* Dex-TA/HA-TA focused on different Dex-TA batches. Figure S4: Viscosity profiles of (a) 10% *w*/*v* Dex-TA/HA-TA and (b) 5% *w*/*v* Dex-TA/HA-TA, focused on Dex-TA batch N/DS 13, with 3 U/mL HRP and different H_2_O_2_/TA molar ratios. Figure S5a: Synthesis of HMW HA-TA conjugates and bioink formation composed of HMW HA-TA conjugates via enzymatic crosslinking. Figure S5b: ^1^H-NMR spectra of HMW HA-TA conjugates: (i) HMW HA-TA had a DS of 5.5% and (ii) HMW HA-TA had a DS of 11%. Figure S6: Shear-thinning property of Pluronic F127 at a polymer concentration of 27.5%(w/v), with and without 0.1% H_2_O_2_. Figure S7: Corresponding confocal fluorescence images of 1.3, 1.8, and 2.2% *w*/*v* BPC-laden core filaments for cell viability on days 0, 1, 3, and 7 (scale bar: 100 μm). Figure S8: Corresponding confocal fluorescence images of 1.3% *w*/*v* 3T3 fibroblast-laden core filaments for cell viability on days 0, 1, 3, and 7 (scale bar: 100 μm). Figure S9: Corresponding bright-field images of 1.3% *w*/*v* 3T3 fibroblast-laden core filaments for cell viability on days 0, 1, 3, and 7 (scale bar: 100 μm).
