# Supplementary material for: Coaxial Bioprinting of Enzymatically Crosslinkable Hyaluronic Acid-Tyramine Bioinks for Tissue Regeneration
Source: Polymers (Basel). 2024 Aug 30;16(17):2470. doi: 10.3390/polym16172470 (PMC11398246; doi:10.3390/polym16172470)
Supplement: Supplementary file 1 [file polymers-16-02470-s001.zip › polymers-3132231-supplementary.pdf]

Supplementary information

1. Synthesis protocols

Synthesis of low and high molecular weight hyaluronic acid-tyramine

Low molecular weight (27 kDa) sodium hyaluronate (5.00 g, 12.5 mmol r.u.) was dissolved in 500 mL Milli-Q water in a 1 L round bottom flask equipped with a stirrer bar. While stirring at room temperature, 4-(4,6-dimethoxy-1,3,5-triazin-2-yl)-4-methylmorpholinium chloride (DMTMM, 3.46 g, 12.5 mmol, 1.0 eq) was added and tyramine hydrochloride (TA·HCl, 2.17 g, 12.5 mmol, 1.0 eq) was added after 30 min. After 24 hours, a saturated aqueous NaCl solution was added slowly to the reaction mixture. After 30 min, the reaction mixture was precipitated in 3 L of cold ethanol. The crude product was thoroughly washed with 250 ml ethanol (x4) and 100 ml Diethyl ether using a Porr 4 filter followed by drying under vacuum for 3 days. The crude product was dissolved in 75 mL Milli-Q water and dialysed against Milli-Q water for 3 days (MWCO 1000 Da) and lyophilization yielded the product as a white foam (5.10 g, 12.4 mmol, 99 % yield, DS 3.8 %. ^{1}H-NMR (400 MHz, D_2O): δ(ppm) = 1.98 (acetyl-CH_3, s, 3H); 2.75 (2-CH_2, s, 2H); 2.90 (1-CH_2, s, 2H); 3.2-4.2 (saccharide ring, m, 10H); 4.34 (s, 1H); 4.43 (d, 1H); 6.84 (Ar m-CH, d, 2H); 7.16 (Ar o-CH, d, 2H).

For high molecular weight (2.0-2.2 MDa) sodium hyaluronate, the same reaction diluted twice (1 L Milli-Q in a 1 L round bottom flask) due to the high viscosity. The procedure can be repeated to obtain a higher degree of substitution. In this case we obtained DS 10 for LMW HA-TA and DS 11 for HMW HA-TA.

The degree of substitution (DS) was calculated based on the integral of the methyl group at 1.98 ppm as compared to the integral of the tyramine signals at 6.80-6.87 and 7.10-7.21 ppm. The DS of hyaluronic acid is given as the percentage of COOH groups modified in hyaluronic acid (i.e. per disaccharide).

Synthesis of dextran-tyramine

Synthesis of dextran-p-nitrophenyl carbonate

LiCl (4.0 g, dried at 115 °C) and dextran (5.00 g, 30.8 mmol repeating units (r.u.)) are weighed into a 500 mL three necked round bottom flask equipped with a stirrer bar. The flask is evacuated and refilled with nitrogen 3 times, after which it is left under vacuum at 95 °C for

1.5h. After thoroughly drying, the flask was filled with nitrogen and 200 mL of anhydrous DMF was added via a cannula while stirring. The flask was then equipped with a thermometer and the mixture was heated to 95 °C while stirring the solution. Once the dextran was completely dissolved, the solution was cooled to 0 °C and anhydrous pyridine (2.6 ml, 33.6 mmol) was added. Subsequently, freshly sublimed para-nitrophenyl chloroformate (3.3 g, 16.4 mmol) was added in small portions, while keeping the temperature below 2 °C. After 1 hour, the reaction mixture was poured into 1 L of ice-cold ethanol. The precipitate was filtered off (Por 4) and washed with cold ethanol (3×100 mL) and subsequently with diethyl ether (3×100 mL). After drying under vacuum, the product was obtained as a white powder (6.31 g, 30.7 mmol r.u., 99% yield, DS25). ^{1}H-NMR (400 MHz, DMSO-d_6): δ(ppm) = 3.0-4.0 (saccharide ring protons, m, 6H); 4.2-5.8 (anomeric and hydroxyl protons, m, 4H); 7.58 (Ar o-CH, d, 2H); 8.34 (Ar m-CH, d, 2H).

Synthesis of dextran-tyramine

Dextran-PNC (6.31 g, 30.7 mmol r.u., 7.67 mmol p-nitrophenyl carbonate) was weighed into a 250 mL three necked round bottom flask equipped with a stirrer bar. The flask was evacuated and refilled with nitrogen 3 times, after which the flask was filled with nitrogen and 120 mL of anhydrous DMF was added via a cannula while stirring. Once the dextran was completely dissolved, tyramine (1.45 g, 10.55 mmol) was added. After 1 hour, the reaction mixture was poured into 1 L of ice-cold ethanol. The precipitate was filtered off (Por 4) and washed with cold ethanol (3×100 mL) and subsequently with diethyl ether (3×100 mL). After drying under vacuum, the crude product was obtained as a white powder. The crude product was dissolved in 75 mL of Milli-Q water and dialysed against Milli-Q water for 3 days (MWCO 3500 Da), followed by filter sterilization and freeze-drying yielding the product as a white foam (5.80 g, 22.2 mmol, 72% yield, DS13.5). ^{1}H-NMR (400 MHz, DMSO-d_6): δ(ppm) = 3.0-4.0 (saccharide ring protons, m, 6H); 4.2-5.8 (anomeric and hydroxyl protons, m, 4H); 6.67 (Ar m-CH, d, 2H); 6.99 (Ar o-CH, d, 2H).

The calculation of the DS of dextran-TA and dextran-PNC is based on the integrals of 4.2-5.8 ppm (corresponding to the 4 anomeric protons from dextran), compared with the integral of the aromatic protons of tyramine (6.60-6.75 and 6.90-7.07) or para-nitrophenyl (7.40-7.65 and 8.20-8.40). The DS of dextran is given as the percentage of saccharide units modified in dextran.

2. Coaxial printing of HMW HA-TA Bioink

a) Bioink preparation (IC)

HMW HA-TA and HRP were dissolved in sterile PBS with a sterile magnetic stirrer to achieve the desired concentration of 1.3- 2.2 %w/v and 5.5 U/ml respectively. Cell pellets were mixed with HMW HA-TA & HRP to reach the final concentration of 1 million cells per ml and to generate a homogeneous mixture before transferring the cell-laden hydrogel into the IC print cartridge.

b) Sacrificial ink preparation (OS)

Pluronic F127 was dissolved in sterile PBS at temperature above room temperature. Pluronic F127 and H_2O_2 were mixed to achieve the desired concentration of 27.5 %w/v and 0.1 % w/v/ respectively and introduced into the OS print cartridge on the day of printing.

c) Bioprinting setup

All equipment, including syringes, coaxial nozzles and the bioprinter chamber, were sterilized following standard sterilization procedures before the day of printing. The bioprinter was set up with coaxial extrusion capabilities and the coaxial nozzles were attached to the syringes containing the bioink and the sacrificial ink. The sacrificial ink print cartridge was connected to the nozzle with the use of a tubing and luer locks.

d) Printing process

G-Code script was uploaded and the bioprinter was priming the nozzles to ensure the smooth flow of both core and shell materials. The printing bed temperature was increased to 30 ºC to avoid gelation of Pluronic F127. The distance between the nozzle and printing substrate was manually adjusted with the Z, Up, and Down arrows (8.0 was used for this experiment). The printing process began by initiating the coaxial deposition of the IC bioink and OS sacrificial ink. A fixed printing speed of 300 mm/min was considered. The different pressures used were as follows: IC pressure between 35-110 kPa for 1.3, 1.8, or 2.2 %w/v of HMW HA-TA. After printing, 1ml PBS was transferred to the petri dish carrying the printed filament for 1min to remove the OS sacrificial material and remaining crosslinking agent. PBS was removed afterward.

Images of printed non-cell-laden core/shell filaments were captured before dissolving Pluronic F127 (for speed match). Images of printed cell-laden core filaments were captured after dissolving Pluronic F127 (to measure the width diameter of the core filaments).

3. G-Code for Coaxial Bioprinting

G21	set units to millimeters.
G90	use absolute coordinates
M83	use relative distances for extrusion
M754	place both extruders in active position
M761	make sure printhead 1 is closed (close extruder 1 valve)
M763	make sure printhead 2 is closed (close extruder 2 valve)
G0 X-10 Y-5 Z0.5 F300	move to start position of first line with speed F300
M760	open printhead 1 (open extruder 1 valve)
M762	open printhead 2 (open extruder 2 valve)
G1 X10 Y-5 F300	
G1 X10 Y-2	
G1 X-10 Y-2	
G1 X-10 Y1	
G1 X10 Y1	
G1 X10 Y4	
G1 X-10 Y4	
G1 X-10 Y7	
G1 X10 Y7	
G1 X10 Y10	
G1 X-10 Y10	
G1 X-7 Y10 Z2	
G1 X-7 Y-10	
G1 X-4 Y-10	
G1 X-4 Y10	
G1 X-1 Y10	
G1 X-1 Y-10	
G1 X2 Y-10	make sure printhead 1 is closed (close extruder 1 valve)
G1 X2 Y10	make sure printhead 2 is closed (close extruder 2 valve)
G1 X5 Y10	
G1 X5 Y-10	
M761	
M763	
G01 Z30	
X0.00 Y0.00	

Supplementary Tables

Table S1: Final concentration of HMW hyaluronic acid-based hydrogels and control (LMW hyaluronic acid/ Dextran) functionalized with tyramine having different degrees of substitution (DS) but with the same enzymatic crosslinking agents.

DS (%)	Polymer concentration (%w/v)	HRP (U/ml)	H_2O_2 (%)
**HMW HA-TA (5.5)**	1.3	5.5	0.0028
	1.8	5.5	0.0038
	2.2	5.5	0.0047
**HMW HA-TA (11)**	1.3	5.5	0.0055
	1.8	5.5	0.0074
	2.2	5.5	0.0092
**LMW HA-TA (10)**	2.5		
**LMW Dex-TA (10)**	2.5	3.0	0.03

Table S2: Optimal rheological properties of pre-crosslinked LMW Dex-TA/HA-TA bioink, printing parameters and results of 3D bioprinting assessment. The microscopic images display the best printing outcome for each bioink considered, corresponding printing parameters and filament spreads (+ standard deviation) are mentioned in the top three rows. A summary of rheological properties is stated in the four rows below. (2x objective). n.a. = data not available. (scale bars: 1 000µm).

	**CELLINK Start**	**Dex-TA / HA-TA 0.033 H_2O_2 / TA**	**Dex-TA / HA-TA 0.040 H_2O_2 / TA**	**Dex-TA / HA-TA 0.044 H_2O_2 / TA**	**Dex-TA / HA-TA 0.047 H_2O_2 / TA**
**Pressure (kPa)**	75 kPa	10 kPa	75 kPa	150 kPa	75 kPa
**Print speed (mm/s)**	25 mm/s	25 mm/s	10 mm/s	25 mm/s	10 mm/s
**Filament spread**	0.93 ± 0.04	3.24 ± 0.49	1.96 ± 0.26	1.55 ± 0.49	1.23 ± 0.23
**Microscopic images**	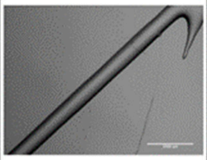	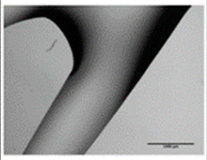	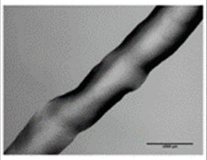	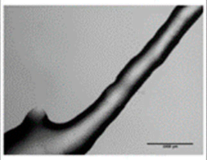	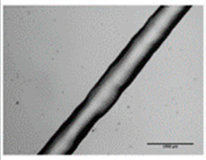
**Viscosity at 10 s^{-1} (Pa·s)**	2.51	0.075	1.89	4.02	4.84
**Flow behaviour index**	0.25	0.74	0.14	0.16	0.19
**Viscosity recovery from shear (%)**	99	*n.a.*	82	40	29
**Yield stress (Pa)**	233	*n.a.*	110	83	352

Supplementary Figures

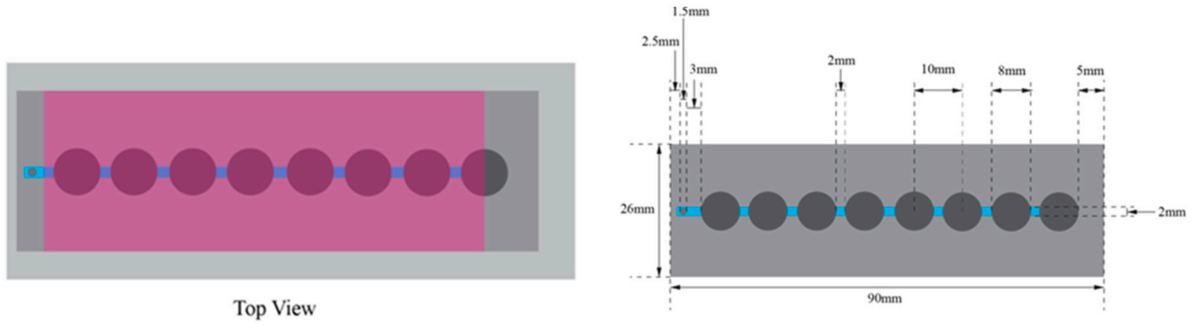

Figure S1: Sketch of the Teflon mold used for hydrogel formation

a)

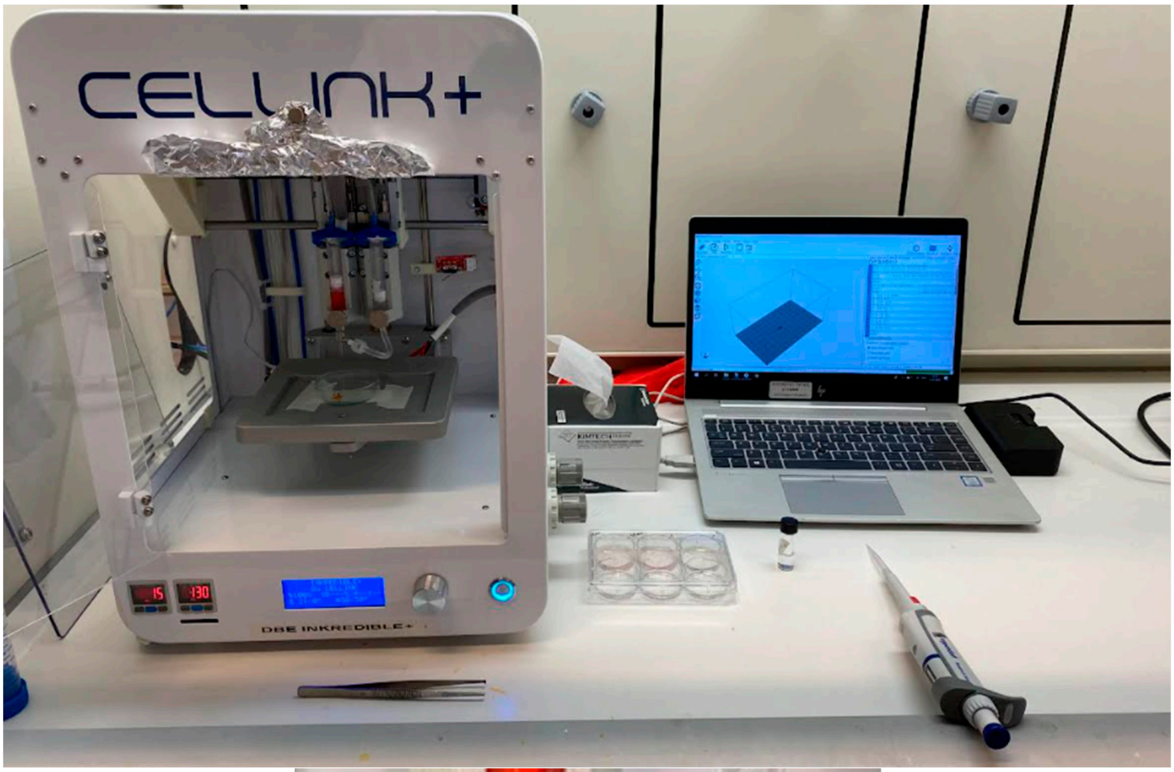

b)

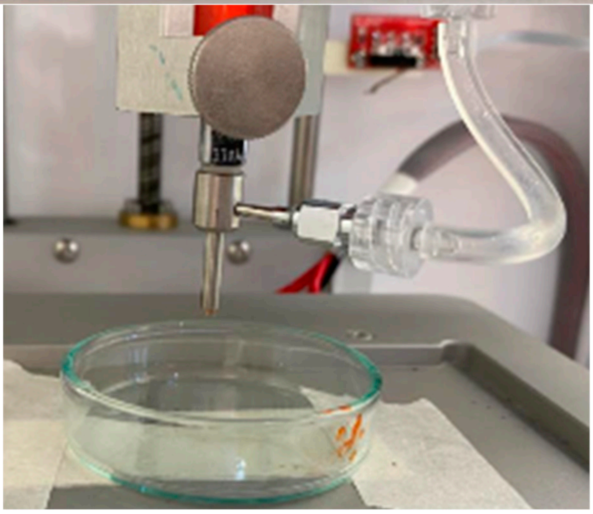

Figure S2a: Inkredible+ 3D Bioprinting system (Cellink) comprising two printheads with independent pneumatic controllers (<150 kPa) and temperature controllers (<40 ^{o}C) **b:** a zoom-in of the coaxial nozzle attached to the Inkredible+ 3D Bioprinting system (Cellink).

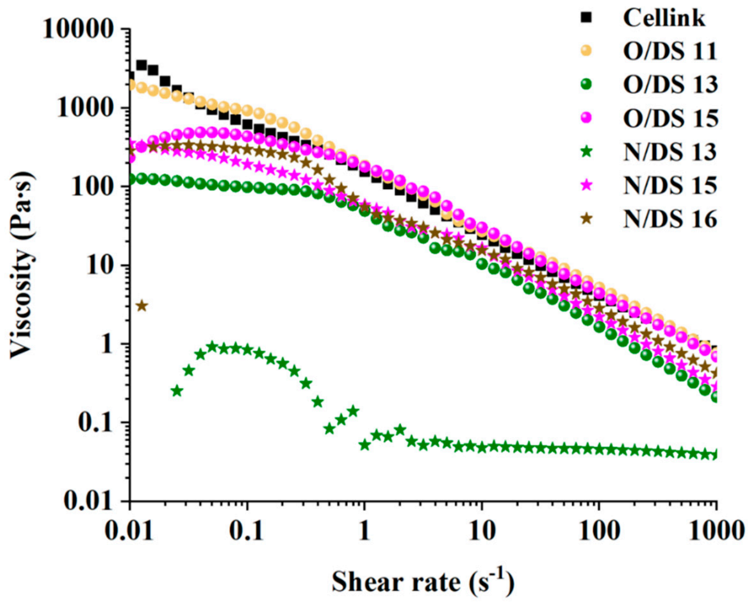

Figure S3: The viscosity profile of 10 %w/v Dex-TA/HA-TA focused on different Dex-TA batches, having 3 U/ml HRP and 0.0041 % H2O2 (0.022-0.028 H2O2/TA). O: old synthesis approach; N: new synthesis approach; DS: degree of substitution. Cellink: Cellink Start bioink. All Dex-TA/HA-TA solutions display viscosity and shear-thinning profiles that are comparable to Cellink Start, excluding the solution with batch N/DS 13. The solution is less viscous and has poor shear-thinning properties.

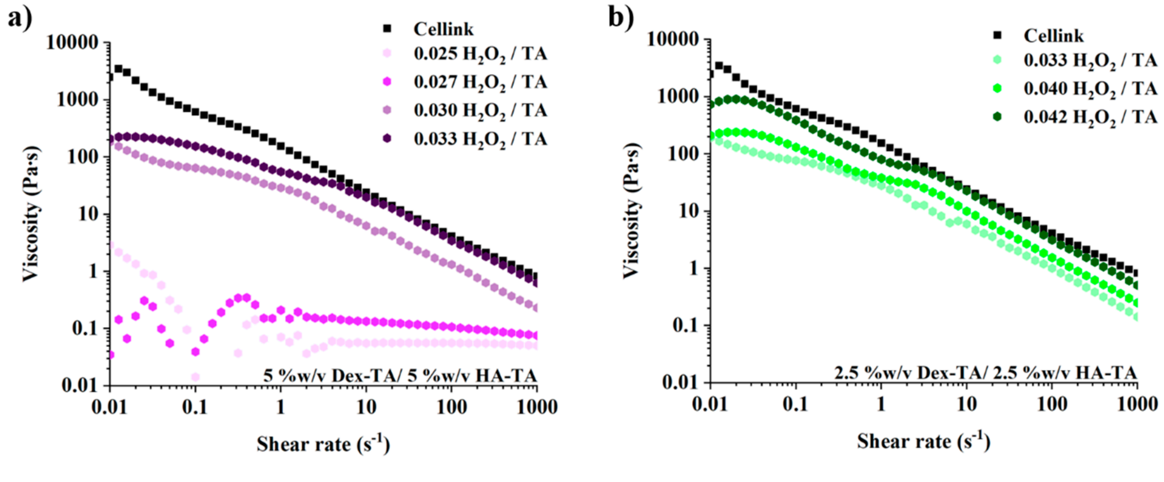

Figure S4: Viscosity profiles a) 10 %w/v Dex-TA/HA-TA b) 5 %w/v Dex-TA/HA-TA focused on Dex-TA batch N/DS 13, having 3 U/ml HRP and different H_2O_2/TA molar ratio. Increase in H_2O_2/TA results to increased viscosity. It is possible to obtain a viscosity profile similar to Cellink Start (black dots). Numbers in legend indicate H_2O_2/TA molar ratio.

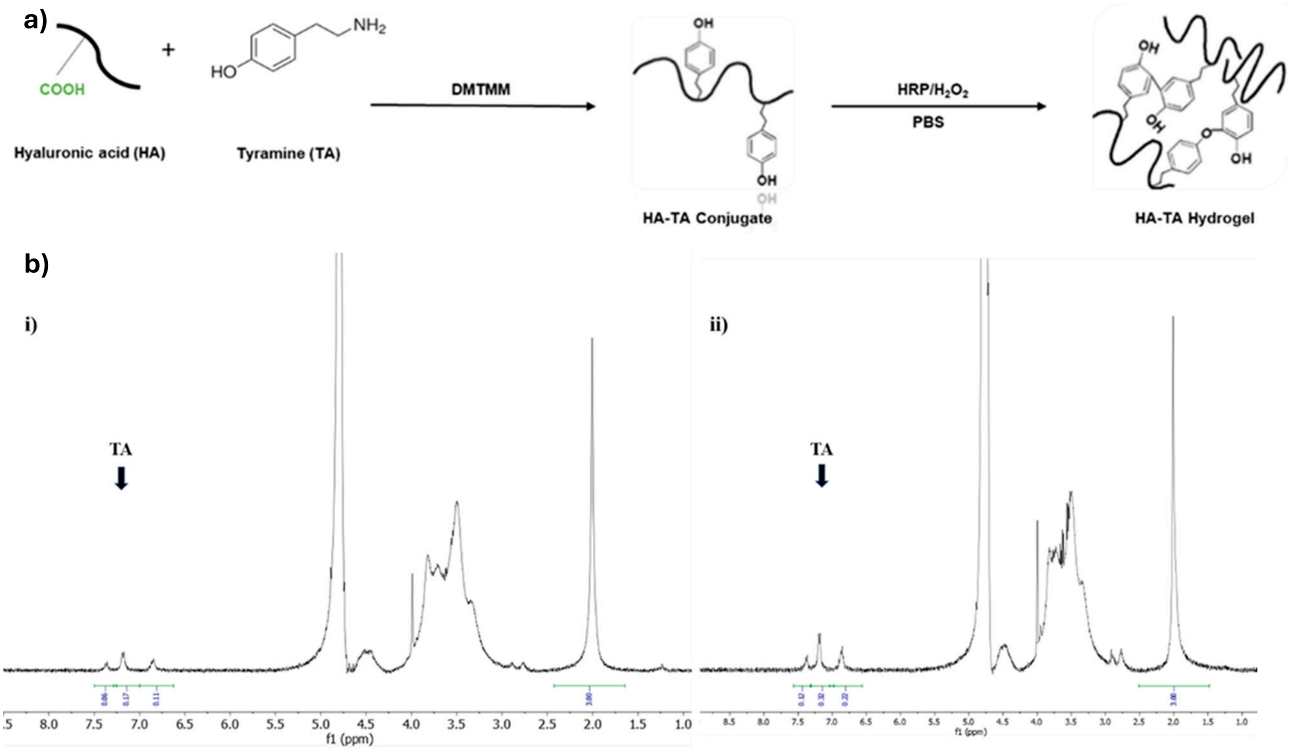

Figure S5a: Synthesis of HMW HA-TA conjugates and bioink formation composed of HMW HA-TA conjugates via enzymatic crosslinking **b:** ^{1}H-NMR spectra of HMW HA-TA conjugates. i) HMW HA-TA had a DS of 5.5% ii) HMW HA-TA had a DS of 11 %.

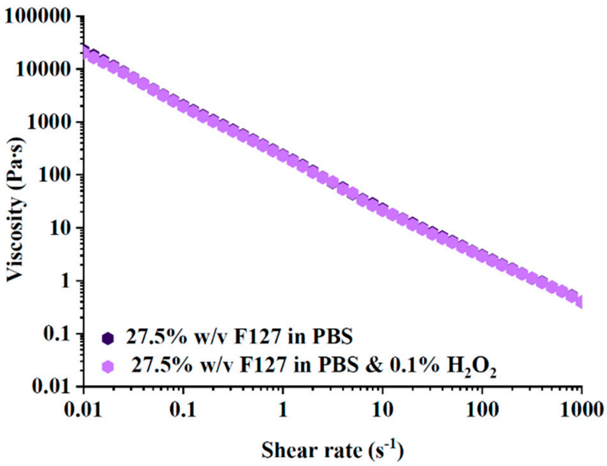

Figure S6: Shear-thinning property of Pluronic F-127 at a polymer concentration of 27.5 %(w/v), with and without 0.1% H_2O_2.

1.3 %w/v HMW HA-TA

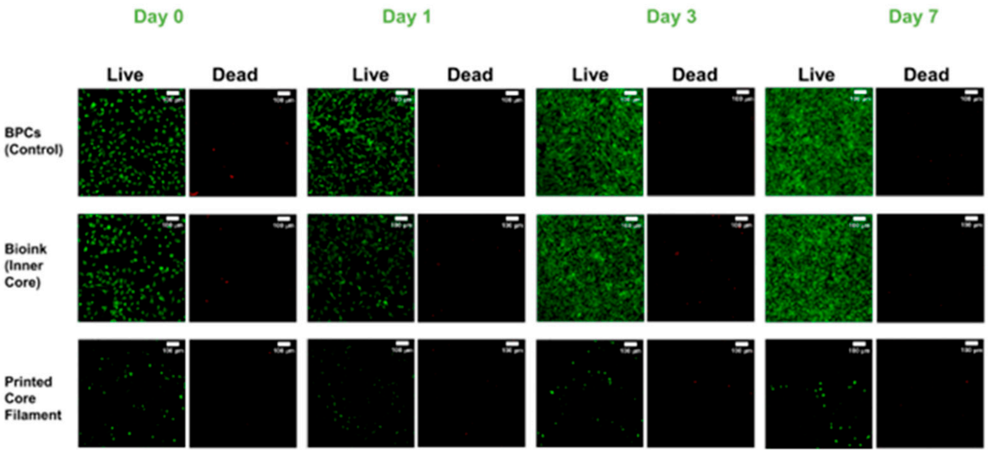

1.8 %w/v HMW HA-TA

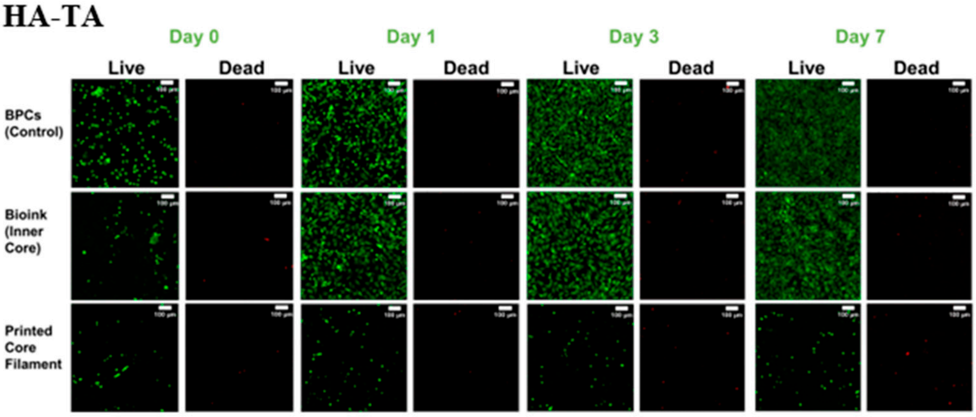

2.2 %w/v HMW HA-TA

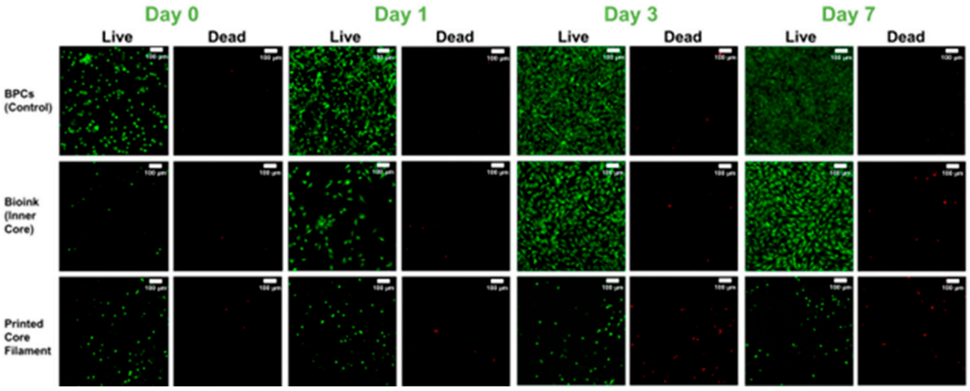

Figure S7: Corresponding confocal fluorescence images of 1.3, 1.8 and 2.2 %w/v BPC-laden core filaments for cell viability on days 0, 1, 3 and 7 (Scale bar: 100 µm).

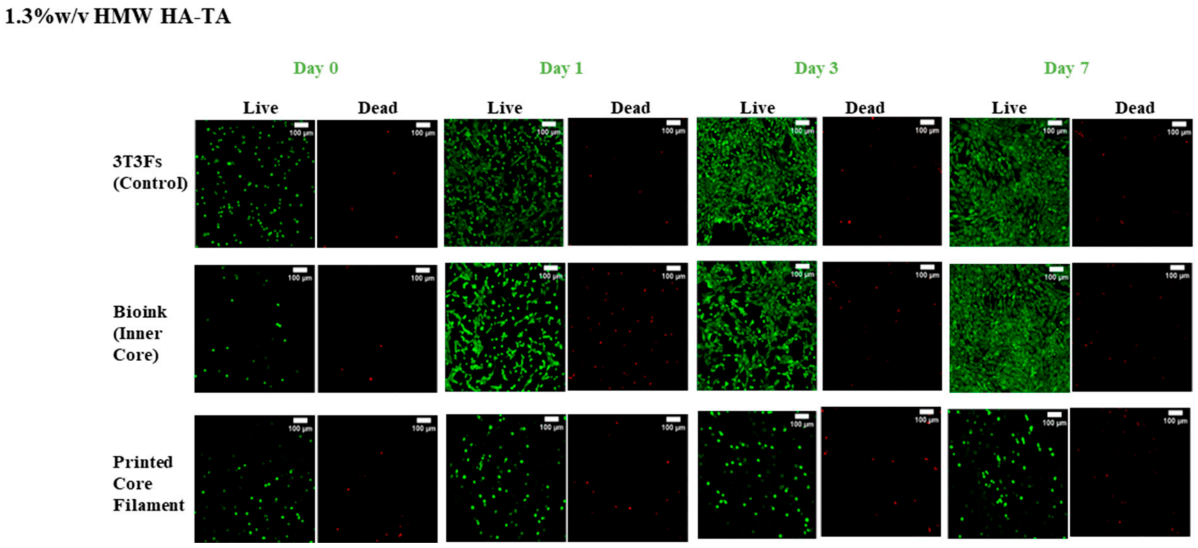

Figure S8: Corresponding confocal fluorescence images of 1.3 %w/v 3T3 Fibroblast laden core filaments for cell viability on days 0, 1, 3 and 7 (Scale bar: 100 µm).

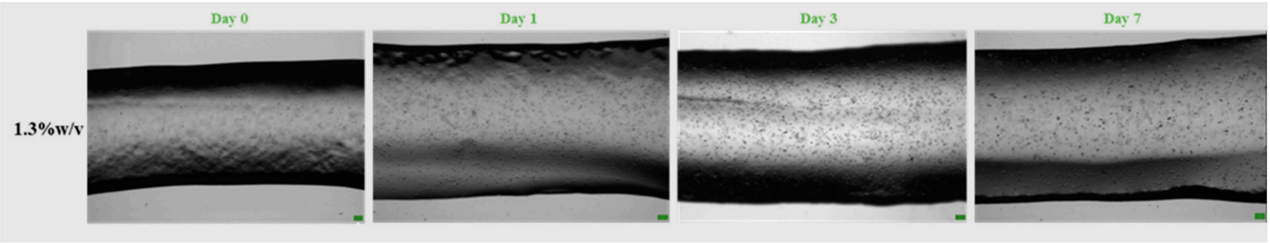

Figure S9: Corresponding bright-field images of 1.3 %w/v 3T3 Fibroblast laden core filaments for cell viability on days 0, 1, 3 and 7 (Scale bar: 100 µm).
